# Targeted HIV Self-Testing Identifies Persons with Undiagnosed HIV and Active Engagement Links them to Care: The GetaKit Study

**DOI:** 10.1007/s10461-024-04302-5

**Published:** 2024-03-25

**Authors:** Patrick O’Byrne, Abigail Kroch, Lauren Orser, Nikki Ho, Alexandra Musten, Marlene Haines, Jennifer Lindsay

**Affiliations:** 1 School of Nursing, University of Ottawa, Ottawa, ON Canada; 2 Ontario HIV Treatment Network, Toronto, ON Canada

**Keywords:** HIV testing, self-testing, Priority populations, Retesting

## Abstract

Current international HIV testing guidelines recommend that HIV negative persons from HIV priority groups complete repeat screening every 3–6 months; local guidelines in our jurisdiction recommend that such retesting should occur every 3 months. Such an approach allows for timely HIV diagnosis and linkage to care – and aligns with the UNAIDS 95-95-95 targets to have 95% of undiagnosed persons be aware of their HIV status. To meet these aims, new approaches to HIV testing have been developed, including our HIV self-testing initiative, GetaKit.ca, which uses an online screening algorithm to determine eligibility and has built in pathways for re-test reminders, linkage HIV prevention care, and rapid follow-up for positive test results. To understand self-testing frequency in relation to our local recommendations for resting every 3 months, we evaluated data from participants who ordered repeat HIV self-tests through GetaKit.ca. Descriptive analyses were performed on participant characteristics and chi-square tests were performed on aggregated participant risk data. During the study period, 5235 HIV self-tests were distributed to 3627 participants, of whom, 26% ordered more than once and 27% belonged to an HIV priority population. Participants who retested were more likely to have been white, male, and part of an HIV priority population; they were also more likely to have completed prior STI or HIV testing or had a prior STI diagnosis, compared to those who did not. We identified 16 new HIV diagnoses, 2 of which were among repeat testers. Our results suggest that HIV self-testing can be useful to help meet UNAIDS targets to identify undiagnosed infections; however, such efforts are less likely to be successful without adequate linkage to follow-up services, including HIV treatment and prevention care.

## Introduction

Guidelines in Canada [1, 2] recommend repeat HIV testing every 3–6 months for persons who both (1) have ongoing risk for HIV acquisition, such as condomless sex with more than one person, the use or sharing of needles or equipment for injecting drugs, engagement in sex work, or experiencing intimate partner violence, and (2) belong to the groups most affected by HIV, henceforth referred to as ‘priority populations’, including gay, bi, trans, and other men who have sex with men (gbtMSM), persons of African, Caribbean, or Black (ACB) ethnicities, members of Indigenous communities, and persons who use drugs (PWUD). The United States Centers for Disease Prevention and Control also recommend repeat testing every 3–6 months for gbtMSM [3]. Locally, in Ontario (where this research occurred), retesting is recommended every 3 months for members of priority populations who have ongoing risk factors for HIV acquisition [2].

The rationale for these testing frequencies in priority populations is that, in some research [4, 5], they corresponded with a shortened time between HIV acquisition and diagnosis – which should limit onward transmission. Indeed, estimates [6, 7] suggest that 50–70% of HIV transmission involves persons with undiagnosed HIV because, in the absence of HIV treatment, viral loads are often elevated making HIV transmission more likely to occur if people continue to engage in the sexual or drug use practices that resulted in their HIV acquisition.

HIV testing is one means to reduce the proportion of persons with undiagnosed HIV infection – and to fulfill the 2030 UNAIDS 95-95-95 targets [8], wherein 95% of persons living with HIV are diagnosed, 95% of those diagnosed are linked to care, and 95% of those linked to care achieve undetectable viral levels. HIV self-testing, as the newest technology, has been heralded as one strategy to help achieve these UNAIDS targets, and this is because observational research shows that it often (1) corresponds with high uptake among HIV priority populations and (2) demonstrates test positivity comparable to or exceeding that of testing done in traditional settings [9, 10].

Notwithstanding the foregoing potential benefits identified in observation studies, the only randomized controlled trial on HIV self-testing identified that, while this testing strategy corresponded with “high HIV testing rates”, it “did not lead to increased rates of new diagnoses” [11, p.e838]. This suggests that while participants liked self-testing, this testing modality did not increase diagnosis or decrease the proportion of persons with undiagnosed infection. Other research has further undermined the potential utility of HIV self-testing due to high rates of invalid test results from these devices (invalid rates ranging from 0.2 to 56.3% [average 6.8%] in unassisted studies) [12]. These findings suggest that self-testing may not be the easiest way to test due to challenges with obtaining sufficient blood or saliva samples, following test instructions, and interpreting results [12]. Another real-world performance shortcoming is the risk of false positive results when a self-test, even one with a high specificity, is used for screening in populations with low HIV prevalence. In a theoretical sample of 1000 persons, an HIV self-test with a specificity of 99.5% would have a positive predictive value of 99.5% in a population with an HIV prevalence of 10%, but a positive predictive value of 16.7% in a population with an HIV prevalence of 0.1%. Appropriately targeting HIV self-tests to those with a higher pretest probability might be ideal. Lastly, false negative results are also a concern if people use these devices during the HIV window period and do not repeat them more than 3 months after a potential exposure to HIV. Taken as a whole, HIV self-testing is a useful addition to achieving the UNAIDS 95-95-95 targets, but not a clinical or public health panacea.

On the side of HIV surveillance, HIV self-testing also raises some new issues. The monitoring of HIV self-testing is challenging, as tests can be purchased by consumers directly without interacting with the healthcare system, and test results are not recorded. Many jurisdictions monitor HIV testing to ensure testing guidelines are met and that high-risk individuals test at appropriate intervals [13]. The inclusion of HIV self-testing creates a challenge for such efforts and signals that we need to explore user behaviour to better understand how self-testing might help achieve the UNAIDS 95-95-95 targets. To understand self-testing behaviour and testing intervals, we reviewed data from GetaKit.ca [14, 15] – an HIV self-testing project in Canada that enables persons to order free HIV self-tests according to guidelines.

## Methods

### Design, Eligibility, and Recruitment

GetaKit.ca is a prospective observational open cohort study that is available for eligible persons. See Table 1 for eligibility criteria. We raised awareness about GetaKit.ca through informational posts and videos on our social media accounts (Instagram, Facebook, X [formerly Twitter]) promoting the availability of self-tests, how the test operates, and how to interpret results. We also ran paid advertisements on Google directing individuals to the site when a search for HIV testing or HIV self-testing was made. Lastly, we worked with local AIDS service organizations, who reposted our social media materials and handed out posters and cards at local events.

**Table 1:** Study eligibility criteria

Eligibility Criteria	Details
• HIV risk factors	• Sexual practices that can transmit HIV (vaginal and/or anal sex) • Injection drug use
• Age	• ≥ 16 years old
• Jurisdiction	• Live in Ontario
• Medication conditions (per Health Canada)	• Not diagnosed with HIV • Do not have a bleeding disorder

### Enrollment and Data Collection

Interested persons accessed GetaKit.ca, where they reviewed and signed the research consent form, registered using their name and contact information (email and/or phone number and mailing address), and completed a sexual health risk assessment, which determined eligibility for testing (Table 1). Onsite registration, which did not require Internet or the disclosure of contact information, was also available at designated agencies.

In all cases, the GetaKit.ca risk assessment collected information each time a participant requested an HIV self-test about their demographics (race/ethnicity, country of birth, sex/gender, sexual orientation), risk practices (sex practices, drug use, sex work), and past medical history (STI/HIV testing/diagnoses, use of HIV pre-exposure prophylaxis [PrEP]). The risk assessment also posed questions about participants’ sexual partners (including if they had transmissible HIV infection, if they used injection drug use, if they were guys who have sex with guys, etc.). The risk assessment also inquired about why people were seeking testing, with answer options including “new sexual partners”, “starting a new relationship”, “outside the window period now”, “my partner has other partners”, and “other” – which allowed for freeform additions that were reviewed by the study team.

GetaKit.ca operated using an algorithm which, first, imputed a risk score based on participants’ answers to the risk assessment questions, and, second, used this risk score to recommend sexual health services, including STI/HIV testing, PrEP, vaccinations, and Naloxone [16]. In short, the risk algorithm ensured that persons had a reason for testing (in that, they had a bona fide risk of potential HIV exposure), including sexual and/or drug use practices. For first-time testers, this included any possible risk factor at any time. Repeat testers needed to report ongoing risk factors for HIV acquisition, such as new sexual partners or new instances of injection drug use; retesting outside the HIV window period was also a permissible reason for retesting. Additional details on the functionality of this algorithm, including assessment questions and diagrams demonstrating how the algorithm determines risk, can be found here [16].

Eligible participants received a fingerstick, blood-based INSTI® HIV self-test – as the only approved device in Canada. Those without risk factors or who were already diagnosed with HIV were ineligible to receive an HIV self-test. Following the United States CDC PrEP guidelines [17], persons who used PrEP and complete quarterly HIV serology were also ineligible.

Results reporting for GetaKit.ca was not required, but participants received a message encouraging them to report a result at approximately 1 and 2 weeks after ordering. Participants could report results by logging into their accounts or by using the ‘public submit’ option, which was a lower barrier method for persons to submit results and receive follow-up. This so-called ‘public submit’ option was available to anyone who obtained an HIV self-test from GetaKit.ca or by other means (e.g., from other self-testing services or if the person had purchased a self-test from the manufacturer or pharmacy). One caveat is that participants had to report a result before re-ordering, but this included ‘prefer not to report’ as an option to select.

Participants who reported results were given tailored information about retesting timelines, information on and referrals for PrEP, and, for those who reported positive results, confirmatory testing and linkage to care from registered nurses. In this way, a person could be eligible for an HIV self-test through GetaKit.ca, then seek PrEP as recommended by the GetaKit.ca system, and subsequently become ineligible for retesting through the study. Participants who belonged to HIV priority populations also received a message 3 months after their order encouraging them to retest if they had new risk practices or if they were in the HIV testing window period during their previous test. A link to reorder was provided in the message.

For any person who reported a positive result, confirmatory testing via serology was advised as a first step in follow-up given the 99.5% specificity of the INSTI® test (meaning false positive results can occur for 5 in 1000 persons), for which, participants were directly referred by a GetaKit.ca nurse to a healthcare provider in their area to complete. Participants also received information regarding HIV management (medical care, treatment), contact tracing for partners who may have been exposed to HIV, mental health counselling, and community support services. We also worked with an HIV legal service to develop messaging about HIV disclosure laws in Canada and provided all persons with a positive self-test result resources for such services to obtain more information and support, including linkage to anonymous HIV confirmatory testing.

### Timeline

We piloted GetaKit.ca starting in July 2020 in Ottawa, Canada and began expanding across Ontario on April 1, 2021. Full availability across Ontario occurred in July 2021.

### Data Analysis

We extracted test orders from the GetaKit.ca database for April 1, 2021-June 2, 2023, and used this dataset to create the analytic period of April 1, 2021-March 31, 2023. Because retesting (per our local guidelines) would have been 3 months after an initial test, the extra time in the dataset we extracted allowed us to include those who ordered at the end of the data collection period as having retested (or not) according to guidelines. We excluded tests ordered during those additional 3 months but classified persons as having ‘re-ordered’ if they placed a new order within that time. The order of tests after the reporting of an invalid result were excluded, as we considered these as replacement orders, not retesting. We reported descriptively on participant characteristics, using means and frequencies. To eliminate small sample sizes (< 5), we aggregated gender into sex (male vs. female), race/ethnicity into white or Black, Indigenous, or Person of Colour (BIPOC), country of birth as Canada or other, and sexual orientation as heterosexual or 2SLGBTQ+. Priority populations were considered people of the following groups [1, 2]: gay, bi, trans, and men who have sex with men, African, Caribbean, or Black people, Indigenous Peoples, and people who use drugs. We performed chi-square tests on these aggregate groups regarding repeat testing using an *a priori* established significance of *p* ≤ 0.05.

### Funding and Ethics

GetaKit.ca was funded by the Ontario HIV Treatment Network (EFP-2020-DC1) and had research ethics board approval from the University of Ottawa (H-12-20-6450). All HIV self-tests, including distribution, were paid for through study funds.

## Results

Between April 1, 2021-March 31, 2023, HIV self-tests were ordered by 3627 unique participants, to whom we distributed 5235 HIV self-tests. These participants had a median age of 30 years, and 78% (*n* = 2821/3627) belonged to HIV priority populations. Most participants were white, cis-male, and 2SLGBTQ+. (Table 2). For sexual health histories, 61% (*n* = 2097/3431) of participants reported prior STI/HIV testing and, of those who had done testing, 30% (*n* = 626/2097) noted a previous diagnosis: 380 reported a prior chlamydia infection, 270 reported a prior gonorrhea infection, and 120 reported a prior syphilis infection. Lastly, 9% (*n* = 293/3437) of participants reported injection drug use and 9% (*n* = 297/3430) reported engaging in sex work.

**Table 2:** Characteristics of self-testing participants

Characteristic		Number	Percentage
Gender (n = 3574)	Cis male	2317	65%
	Cis female	871	24%
	Trans male	65	2%
	Trans female	46	1%
	Nonbinary	244	7%
Sexual orientation (n = 3495)	2SLGBTQ+	2259	65%
	Heterosexual	1047	30%
Ethnicity (n = 3600)	White	1424	40%
	BIPOC	1934	60%
	ACB	642	18%
	South East Asian	529	15%
	South Asian	262	7%
	Latino/a/x/e	183	5%
	Middle Eastern	178	5%
	Indigenous	98	3%

Next, 26% (*n* = 928/3627) of participants ordered at least twice, and these participants ordered 48% (*n* = 2536/5235) of tests distributed during the study period. Retesting occurred among 27% (*n* = 772/2841) of those who were members of priority populations, compared to 19% (*n* = 156/820) who did not belong to a priority population. These 156 non-priority population participants used 393 self-tests, versus re-testers in the priority populations who used 2143 tests.

An additional 567 people sought to reorder 819 times, but were deemed ineligible by the GetaKit.ca algorithm; 15% (*n* = 124/819) of these orders were deemed ineligible because participants initiated PrEP since their prior order; the remainder were ineligible because participants requested retesting too frequently (less than every 6 weeks) or did not report new risk factors for HIV acquisition after previously testing outside the window period at their last order.

Comparing those who had retested versus had not, we found significant differences. (Table 3). Participants who retested were more likely to have belonged to a priority population than not; they were more likely to be male compared to female; they were more likely to be gbtMSM compared to heterosexual; participants who retested were also more likely to have a reported history of STI/HIV testing and were more likely to have reported a past STI diagnosis, versus no diagnosis. Participants who reordered were more likely to have been white compared to ACB or Indigenous, although no differences were identified for white compared to BIPOC participants overall. There were no significant differences between participants with single or repeat testing among those who reported speaking English as a first language or not, being born in Canada or elsewhere, engaging in sex work or not, or using injection drugs or not.

**Table 3:** Chi-square tests comparing participant characteristics according to testing behaviour

Characteristics		Testing		*X* ^*2*^	p-value
		Single	Repeat		
Priority population	Yes	772	2049	21.13	< 0.001
	No	156	650		
Sex	Male	450	1412	12.56	< 0.001
	Female	137	631		
Sexual orientation	gbtMSM	550	1137	26.1	< 0.001
	Hetero	229	818		
Prior STI testing	Yes	618	1479	48.78	0.011
	No	251	1082		
Prior STI diagnosis	Yes	175	1608	220.8	< 0.001
	No	450	1032		
Ethnicity	White	387	1037	1.7	NS
	BIPOC	487	1447		
	White	387	1037	6.45	0.011
	ACB or Indigenous	164	576		
Language	English	908	2629	0.75	NS
	Other	19	69		
Country of birth	Canada	517	1391	0.11	NS
	Other	335	876		
Sex work	Yes	67	230	0.72	NS
	No	802	2435		
Injection drug use	Yes	66	227	1.34	NS
	No	805	2339		

Next, 16 participants reported positive results to GetaKit.ca during the study period. These persons were white (*n* = 6) and BIPOC (*n* = 10). Thirteen of these participants were male (all with same sex partners) and three were female. Participants who reported positive results were more likely to have ordered only once and to have tested positive on their first order (*X*^*2*^ 3.52 *p* = 0.06). Indeed, 88% (*n* = 14/16) of the positive HIV self-test results reported to GetaKit.ca during the study occurred on the first test ordered, and 50% (*n* = 8/16) of participants reported that this was their first instance undergoing HIV testing at all. Only two positive results were reported among those who ordered more than once. Among those who reported prior HIV testing, 50% (*n* = 4/8) stated that this testing was more than 12 months ago. Therefore, only 25% (*n* = 4/16) of persons who reported positive self-test results to GetaKit.ca had completed HIV testing within the previous 12 months, despite all having risk factors that would recommend HIV testing every 3 months in our jurisdiction.

The overall positivity rate was 0.4% for all GetaKit participants (*n* = 16/3627) and 0.3% for tests distributed (*n* = 16/5235). The positivity rate for one-time testers was 0.5% (*n* = 14/2699), whereas the positivity rate for repeat testers was 0.2% (*n* = 2/928) when calculated for the number of participants who re-tested, and 0.08% (*n* = 2/2536) when calculated for the total number of tests distributed to repeat testers. All 16 participants who reported positive results responded to follow-up within 48 h and were linked to care. Only 1 participant had initiated linkage to care on their own before follow-up, while the remaining were uncertain regarding next steps for confirmatory testing and only began linkage-to-care and follow-up once we directly provided such services.

## Discussion

Herein, we reported on the 3627 participants who obtained HIV self-tests from GetaKit.ca between April 1, 2021-March 31, 2023. We identified that one-quarter of these participants retested and that participants who retested were more likely to be gbtMSM and previously tested, but less likely to be ACB or Indigenous, compared to white, although these differences disappeared when we expanded our analysis to all BIPOC participants. Furthermore, most of the participants who reported a positive HIV self-test result did so on their first test, and half of our participants who reported a positive self-test result indicated that this was their first HIV test ever. Repeat testing, in contrast, only yielded two additional diagnoses, despite accounting for half of the HIV self-tests we distributed. These findings raise a few important points for discussion.

First, HIV self-testing in our study provided access to members of HIV priority populations [1], first-time testers [1], and persons with undiagnosed HIV infections [8], yielding a positivity rate of 0.3% (all tests), 0.4% (all GetaKit participants), and 0.5% (all participants’ first test). These rates match focused testing initiatives in our jurisdiction (Ontario, Canada) and exceed the 0.1% positivity rate seen in traditional clinical settings [18]. These findings highlight the role of GetaKit.ca in providing testing to, and linkage to care for, persons with undiagnosed HIV infection who have not previously or who have not recently done testing.

These findings, moreover, provide real-world data from Canada to support the assertion that HIV self-testing can help achieve the UNAIDS 95-95-95 targets [8] – but with caveats. On the one hand, our positivity rates were likely elevated (compared to laboratory-based testing) because we targeted our distribution of self-tests to those at higher risk using the GetaKit.ca algorithm [16]. Mass distribution of self-tests might not achieve the same diagnostic outcomes and positivity rates [11, 19], and could ultimately waste resources without identifying new infections. On the other hand, although we achieved a 100% linkage-to-care rate, this was only because our program was designed and funded to collect key information from, and establish an ongoing connection with, people who obtained HIV self-tests from us. Indeed, in advance of launching GetaKit.ca, we created pathways which included formal relationships with treatment centres where GetaKit.ca participants could do follow-up in the event of a positive test. We also created a ‘public submit’ reporting mechanism through which participants could easily report results and obtain follow-up from the study team. Our 100% linkage-to-care rate also only occurred because nurses from the study team rapidly reached out by phone to all persons who reported positive results to provide support and to ensure linkage occurred, including confirmatory testing and treatment [20].

One cannot assume, therefore, that self-testing without wrap-around supports would yield the same outcomes. In our study, only one participant who reported a positive self-test result had attempted to initiate linkage to care before we directly reached out to them, suggesting that linkage-to-care may not occur or may be delayed if self-testing initiates (1) do not send reminders about, or simplify the process for, results reporting, and (2) do not initiate contact with persons who report positive results. While self-initiated strategies on the part of testers, such as text or instant messaging platforms [19] or telephone call centers [21], can be used to facilitate linkage to care for persons who receive a positive HIV self-test, research has demonstrated that personal outreach, including direct linkage to health and social supports, corresponded with better retention rates for HIV treatment and HIV care [22, 23], compared to those who have to seek out confirmatory testing, treatment, and supports on their own [19, 21]. Those who intend to make self-testing available should likely heed our strategy, and, before implementing this intervention, establish care pathways. Otherwise, while self-testing may help achieve the first 95-95-95 target to increase diagnosis rates, there is no guarantee it will contribute to the next two targets for linkage-to-care and viral suppression [8]. There are also ethical issues to offering testing without supporting those who test positive, and it is important to consider that, in some jurisdictions, diagnosis through a self-test may imperil individuals if they were to acquire a legal duty to disclose their HIV-status [24] – particularly if the person has transmissible (untreated) HIV. Offering HIV self-tests without linkage-to-care may inadvertently exacerbate inequities, therefore, if persons who completed this testing do so without being informed about the risks associated with testing positive.

Second, HIV self-testing initiatives must also focus on linking persons to prevention services [17]. Increased testing without corresponding increases in prevention will not necessarily reduce the number of persons with undiagnosed infections [25], as persons would continue to acquire HIV at the same rate. In other words, any effect from testing would only last for as long as intensified testing initiatives were maintained. As we observed with GetaKit.ca, self-testing programs can contribute to prevention efforts by developing pathways that promote and link persons to PrEP, when indicated [17]. Our data showed that at least 124 persons – or 10% of those who sought retesting – initiated PrEP after accessing the study for the first time. This finding thus suggests that HIV testing can promote both diagnosis and PrEP, especially when automated algorithms recommend clinically indicated services based on participants’ reported results.

Third, retesting among GetaKit.ca participants was more common among members of HIV priority populations, which could have been the result of the retest reminders we sent to these participants or due to pre-established group norms about HIV testing within these populations. While a randomized controlled trial is the only way to answer this question, evidence from cancer screening initiatives [26, 27] (as the only evidence on this topic) suggests that retest reminders link people back to screening at clinically recommended intervals. In the absence of harms from promoting retesting, we encourage the adoption of retest reminders to promote engagement in testing. Although we only identified 2 new infections among those who retested (versus 14 among those who obtained only 1 test), any identification of, and linkage to care for, a new HIV infection has both individual and population health benefits, regarding improved health status and decreased transmissibility, respectively.

Retesting in our study, however, was not equally distributed. Although we did not identify differences in retesting rates among BIPOC participants compared to white participants, we did observe lower retesting rates among ACB and Indigenous participants. This marks an area for improvement, as the UNAIDS 95-95-95 targets indicate “95% of people within the sub-populations who are living with HIV know their HIV status” [8, p.1]. While the 90-90-90 targets were based on overall rates, to achieve the 95-95-95 targets, a 95% diagnosis rate must be achieved in each population [8]. Lower rates of diagnosis and engagement in treatment in some populations [28], such as Indigenous peoples, can no longer be effaced by higher rates in other populations with better access to services. Efforts must be made to ensure increased testing and linkage to treatment for all communities. GetaKit.ca data suggest that increasing routine retesting in racialized populations may prove challenging, but that self-testing appears to be one successful strategy. Indeed, over one-fifth of GetaKit.ca participants identified as ACB or Indigenous, and nearly two-thirds of our new diagnoses identified as BIPOC – although this might have been skewed by our promotion through AIDS service organizations that work with and for these communities. Self-testing thus plays a role, even if further efforts are required to improve how it functions.

### Limitations

Our results must be interpreted considering certain limitations. First, they may not be generalizable to the general population or to all people at risk for HIV. For one, our data had low rates of trans participants, thus limiting applicability to this population. As well, these data arose from a system that is primarily web-based, meaning that our findings may be skewed based on those who can access the Internet. However, it is of note that all positive results during the study period arose from persons who used the online system; none from those who accessed HIV self-tests from us through partner agencies. Further, GetaKit.ca eligibility is linked to HIV risk and therefore those individuals who are not eligible are not as likely to further interact with the program. Second, 60% of GetaKit.ca participants reported their results back, and some of the unreported results may be positive, impacting our interpretation of follow-up and test positivity. While financial incentives (e.g., money, gift cards) could have been used to increase results reporting, we opted against this due to ethical concerns of requiring participants to disclose their results for payment – particularly if this result was to be positive and the person might not have otherwise disclosed. It is also possible that participants gave the HIV self-test(s) they obtained from our study to others (e.g., partners or friends) and may not have even known the test results themselves. We, however, have no evidence that this did or did not occur – which warrants further research about onward distribution of HIV self-tests within HIV priority populations. Third, these data represent the minimum HIV retesting rates in our jurisdiction. Participants may have done a non-GetaKit.ca self-test, an HIV point-of-care test, or serology after their GetaKit.ca self-test (including having received a positive result), which would not have been captured in our data. Further, those who were linked to PrEP were no longer eligible for self-testing and should therefore no longer order through GetaKit.ca for their testing, indicating a successful linkage and not a failure to retest.

## Conclusion

Self-testing is the newest advancement in HIV diagnostics and research shows that it has good user satisfaction, uptake, and positivity rates. For these reasons, efforts have been made to increase access to this testing modality. To determine how self-testing may contribute to HIV retesting, we reviewed the GetaKit.ca database from April 1, 2021-March 31, 2023. While GetaKit.ca demonstrated a test positivity rate which exceeded that seen in traditional healthcare settings in our jurisdiction – and that it was particularly successful in identifying undiagnosed HIV infection in persons who had never previously done testing – it was not without limitations. Retesting using self-tests was not undertaken frequently, and many people used nearly 50% of our self-tests to retest without identifying many additional HIV diagnoses. We also saw lower rates of retesting among participants who identified as ACB or Indigenous. Knowing that the 2030 UNAIDS 95-95-95 targets focus on achieving a 95% diagnosis rate in the general population and among all subpopulations, efforts must now be made to determine how and why retesting rates were lower in these populations. Furthermore, self-testing must be established for everyone such that it ensures linkage to care – whether to prevention or treatment. Without supporting persons with diagnosed HIV to become non-infectious (through undetectable viral loads) and HIV-negative persons non-susceptible to infection acquisition (through PrEP), testing alone through any combination of modalities will likely never decrease the proportion of persons with undiagnosed HIV infection. As we move toward the 2030 date of the 95-95-95 targets, it is important for clinicians, public health workers, researchers, and decision-makers to engage and embed status neutral linkages into self-testing programs. In the absence of such foresight and efforts, we may squander the benefits self-testing could hold to achieve the UNAIDS 95-95-95 in time.

## References

[1] PHAC. HIV Screening and Testing Guide. Accessed on June 11 2023 from https://www.canada.ca/en/public-health/services/hiv-aids/hiv-screening-testing-guide.html.

[2] Ontario. Ontario Guidelines for Providers Offering HIV Testing. Accessed June 11 2023 from https://hivtestingontario.ca/ontario-guidelines-for-providers-offering-hiv-testing/.

[3] Treatment Guidelines CDCSTI. 2021. Accessed on June 11 2023 from https://www.cdc.gov/std/treatment-guidelines/default.htm.

[4] Branson BM, Handsfield HH, Lampe MA et al. Revised recommendations for HIV testing of adults, adolescents, and pregnant women in health-care settings. MMWR 2006; 55(RR-14). 16988643

[5] DiNenno EA, Prejean J, Irwin K (2017). Recommendations for HIV screening of gay, bisexual, and other men who have sex with men – United States, 2017. MMWR.

[6] Marks G, Crepaz N, Janssen RS (2005). Estimating sexual transmission of HIV from persons aware and unaware that they are infected with the virus in the USA. AIDS.

[7] Marks G, Crepaz N, Senterfitt JW, Janssen RS (2005). Meta-analysis of high-risk sexual behaviour in persons aware and unaware they are infection with HIV in the United States: implications for HIV prevention programs. JAIDS.

[8] 2025 UNAIDS, Targets AIDS. Accessed on June 11 2023 from https://www.unaids.org/sites/default/files/2025-AIDS-Targets_en.pdf.

[9] O’Byrne P (2021). HIV self-testing: a review and analysis to guide HIV prevention policy. Public Health Nurs.

[10] O’Byrne P, Musten A (2023). HIV self-testing: what GetaKit can tell us about Canada’s $8 million one-time investment. Can J Public Health.

[11] Rodger JA, McCabe L, Phillips AN, Lampe FC, Burns F, Ward D (2022). Free HIV self-test for identification and linkage to care of previously undetected HIV infection in men who have sex with men in England and Wales (SELPHI): an open-label internet-based randomised controlled trial. Lancet HIV.

[12] Figueroa C, Johnson C, Ford N, Sands A, Dalal S (2018). Reliability of HIV rapid diagnostic tests for self-testing compared with testing by health-care workers: a systematic review and meta-analysis. Lancet HIV.

[13] Ontario HIV, Epidemiology and Surveillance Initiative [OHESI]. HIV tests in Ontario, 2020. 2022. Accessed on June 11 2023 from https://www.ohesi.ca/wp-content/uploads/2022/06/OHESI-report-HIV-tests-in-Ontario-2020.pdf.

[14] O’Byrne P, Musten A, Orser L, Inamdar G, Grayson MO, Jones C, Francoeur M, Lachance S, Paulin V (2021). At-home HIV self-testing during COVID: implementing the GetaKit project in Ottawa. Can J Public Health.

[15] O’Byrne P, Musten A, Vandyk A, Ho N, Orser L, Haines M, Paulin V (2021). HIV self-testing in Ottawa, Canada used by persons at risk for HIV: the GetaKit Study. Can Commun Dis Rep.

[16] O’Byrne P, Musten A, Orser L, Buckinham S (2021). Automated STI/HIV risk assessments: testing an online clinical algorithm in Ottawa, Canada. Int J STD/AIDS.

[17] CDC. Preexposure prophylaxis for the prevention of HIV infection in the United States – 2021 Update. Accessed on June 11 2023 from https://www.cdc.gov/hiv/pdf/risk/prep/cdc-hiv-prep-guidelines-2021.pdf.

[18] OHESI. HIV diagnoses in Ontario., 2020. 2022. Accessed on June 11 2023 from https://www.ohesi.ca/wp-content/uploads/2022/08/HIV-diagnoses-in-Ontario-2020-REPORT-FINAL-1.pdf.

[19] Edelstein ZR, Wahnich A, Purpura LJ, Salcuni PM, Tsoi BW (2020). Five waves of an online HIV Self-Test Giveaway in New York City, 2015–2018. Sex Transm Dis.

[20] International Advisory Panel on HIV Care Continuum Optimization [IAPAC]. IAPAC guidelines for optimizing the HIV care continuum for adults and adolescents. J Int Assoc Provid AIDS Care. 2015;Suppl13–S34. 10.1177/2325957415613442. 10.1177/2325957415613442 26527218

[21] D’Angelo AB, Morrison CA, Lopez-Rios J, MacCrate CJ, Pantalone DW (2021). Experiences receiving HIV-Positive results by phone: acceptability and implications for clinical and behavioral research. AIDS Behav.

[22] Kelly JD, Hartman C, Graham J, Kallen MA, Giordano TP (2014). Social Support as a predictor of early diagnosis, linkage, Retention, and adherence to HIV Care: results from the steps Study. JANAC.

[23] Carballo–Diéguez A, Giguere R, Balán IC, Brown W, Dolezal C (2020). Use of Rapid HIV self–test to screen potential sexual partners: results of the ISUM Study. AIDS Behav.

[24] Canadian HIVAIDS, Legal Network. The criminalization of HIV non-disclosure in Canada: Current status and the need for change. 2019. Accessed 11 June 2023 from 2949-CanadianHIVAIDSLegalNetwork_HIVCriminalizationDocument-English-061119_FINAL.pdf.

[25] Gray RT, Prestage GP, Down I, Ghaus MH, Hoare A (2013). Increased HIV testing will modestly reduce HIV incidence among gay men in NSW and would be acceptable if HIV testing becomes more convenient. PLoS ONE.

[26] Tsoa E, Pefoyo AJK, Tsiplova K, Kupets R (2017). Evaluation of recall and reminder letters on retention rates in an organized cervical cancer screening program. JOGC.

[27] MacLaughlin KL, Kessler ME, Elayavilli RK, Hickey BC (2018). Impact of patient reminders on Papanicolaou test completion for high-risk patients identified by a clinical decision support system. J Women’s Health.

[28] Rachlis B, Burchell AN, Gardner S, Light L, Raboud J (2017). Social determinants of health and retention in HIV care in a clinical cohort in Ontario, Canada. AIDS Care.

